# Agreement of Middle brook 7H10 with Lowenstein Jensen and accuracy of the Sensititre MYCOTB plate using either method as a reference standard for *Mycobacterium tuberculosis* first line drug susceptibility testing

**DOI:** 10.1371/journal.pone.0199638

**Published:** 2018-06-28

**Authors:** Willy Ssengooba, Germine Nakayita, Carolyn C. Namaganda, Moses L. Joloba

**Affiliations:** Makerere University, Department of Medical Microbiology, Mycobacteriology (BSL-3) Laboratory, Kampala, Uganda; Indian Institute of Technology Delhi, INDIA

## Abstract

**Introduction:**

Although Sensititre *Mycobacterium tuberculosis* (MYCOTB) plate offers both drug susceptibility testing (DST) and minimum inhibitory concentration (MIC) results, it has not been evaluated against both Lowenstein Jensen (LJ) and Middlebrook 7H10 (MB7H10) DST methods at standard critical concentrations.

**Materials and methods:**

We analyzed 76 *M*. *tuberculosis* isolates consisting of 54 isolates from the Uganda National TB drug resistance survey done December 2009–February 2011 and 22 isolates from the World Health Organization External Quality Assessment panel for the year 2011. All isolates were tested for LJ, MB7H10 and MYCOTB plate based DSTs for streptomycin, isoniazid, rifampicin and ethambutol anti-tuberculosis drugs. The agreement of MB7H10 with LJ and accuracy of MYCOTB plate using either LJ-DST or MB7H10 as a reference standard were determined.

**Results:**

The agreement (kappa) of MB7H10 with LJ was; 0.687 for rifampicin, 0.498 for isoniazid, 0.275 for streptomycin and 0.082 for ethambutol which as almost similar when compared with MYCOTB plate. The sensitivity (95% confidence interval; CI) of MYCOTB plate when LJ was used as a reference standard was higher for streptomycin 87.5% (81.6–98.4) followed by isoniazid 75.9% (65.1–95.6) and rifampicin 73.1% (52.2–88.4). When MB7H10 was used as reference standard, the sensitivity of MYCOTB plate improved significantly; isoniazid 96.2% (80.3–99.9), rifampicin 94.0 (83.4–98.7) and 93.8% (69.7–99.8). There was good agreement between MYCOTB plate and MB7H10; 1.00 for ethambutol, 0.959 for streptomycin, 0.915 for rifampicin and 0.778 for isoniazid.

**Conclusions:**

The performance of the two culture-based reference standards for phenotypic first-line drug susceptibility testing methods, LJ and MB7H10, varied much even with acceptable MYCOTB plate MICs. There was acceptable agreement and accuracy of MYCOTB plate for drug susceptibility testing when MB7H10 was used as reference standard than with LJ-DST. Results from MIC information makes the MYCOTB plate more suitable for guiding clinicians on the choice of the most appropriate TB treatment regimen as well as limits of detection for TB drug resistance.

## Introduction

The global burden of tuberculosis has been worsened by the increasing rates of multi-drug resistant (MDR-TB) and extensively drug-resistant tuberculosis (XDR-TB). Based on the results of the 2011 Uganda national drug resistant TB prevalence survey, the burden (95%CI) of MDR-TB and RR-TB in Uganda is 1.6 (0.78–2.4) per 100 000 population among new cases and 12 (5.9–18) per 100 000 population among previously treated TB cases. And according to the World Health Organization report 2016, the incidence of MDR-TB/RR-TB in 2015 was 1.9 (1–2.8) per 100,000 population among new cases and 4.9 (2.6–7.2) per 100,000 population among previously treated TB cases[1]. This indicates an increasing burden of MDR-TB in Uganda and calls for universal testing for drug resistance among all MDR-TB suspects.

Drug susceptibility testing (DST) using the Lowenstein Jensen (LJ) proportional method remains the mostly used phenotypic DST method at the Uganda’s national TB reference laboratory with other methods such as Middlebrook 7H10 (MB7H10) and liquid culture-based DSTs being used in research laboratories. This method has high skills demand and require 4–6 weeks to report the results. Although of recently WHO recommended rapid molecular DST methods; the GeneXpert (Xpert MTB/RIF assay) and Hain Genotype MDRplus and MTBDRsl line probe assay, these methods are also facing performance and implementation challenges. For example, they both have a low coverage regarding drug resistance-conferring mutations as few drugs are included in their panels[2]. A recent study found the Hain MTBDRsl assay to detect synonymous and lineage associated mutations that are not resistance-conferring [3].

These limitations together with the increasing disagreement between phenotypic and rapid genotypic DST methods makes the well-studied phenotypic methods still relevant in the management and control of drug-resistant TB. Some of the discordances are due to borderline resistance or resistance due to different critical concentrations used for DST setting. To resolve such disagreements, determination of the Minimum inhibitory concentrations (MIC) is needed. Clinicians could use the MICs for individualized patient care which may include increasing the dosage or change drugs within a drug class. Determining MICs using conventional proportional methods is technically more challenging. The Sensititre *Mycobacterium tuberculosis* (MYCOTB) plate (TREK Diagnostic Systems, Thermo Fisher Scientific, US) determines the MICs including borderline resistance for various drugs. It is a 96 well plate with twelve lyophilized drugs including first and second line drugs. The test requires minimal training, user-friendly, easy to set and to interpret. However, the MYCOTB plate has not been evaluated concurrently with the two commonly used reference DST methods. The performance of *M*. *tuberculosis* susceptibility testing has been found to differ by platform and by drug[4]. Therefore, set to evaluate the agreement of MB7H10 with LJ and accuracy of MYCOTB plate using either LJ-DST or MB7H10 as a reference standard.

## Materials and methods

### Mycobacterium tuberculosis strains

We evaluated 76 *M*. *tuberculosis* isolates. A total of 44 isolates were from the Uganda National TB drug resistance survey of December 2009–February 2011, ten isolates were from routine testing and 22 isolates were from the World Health Organization External Quality Assessment (WHOEQA) panel for the year 2011.

### Drug susceptibility testing using Lowenstein Jensen media

The LJ-DST were performed for streptomycin, isoniazid rifampicin and ethambutol drugs at the National Tb reference laboratory (now the WHO Supra-National TB reference Laboratory; SRL in Uganda). Briefly, isolates were tested using the standard drug concentrations for rifampicin (40 mg/mL), isoniazid (0.2 μg/ml) and streptomycin (4 μg/ml) for which results were interpreted at week six, and for ethambutol (2 μg/ml) which was interpreted at week four as previously described [5].

### MB7H10 media-based drug susceptibility testing

The culture plates for MB7H10 (*M0303 Sigma-Aldrich*) were prepared in-house following the guidelines described as per the Clinical and Laboratory Standards Institutes (CLSI) methods guidelines for indirect DST as described elsewhere[6]. These were performed at the Mycobacteriology (BSL-3) laboratory of the Department of Medical Microbiology, Makerere University, Kampala, Uganda. The critical concentrations (μg/mL) for the first line drugs were: streptomycin 2 and 10, isoniazid 0.2 and 1, rifampicin 1 and ethambutol 5 and 10. Plates were incubated at 37^0^c under 5% Carbon dioxide for 21 days prior to results interpretation.

### Drug susceptibility testing using MYCOTB plate

**Fig 1:**
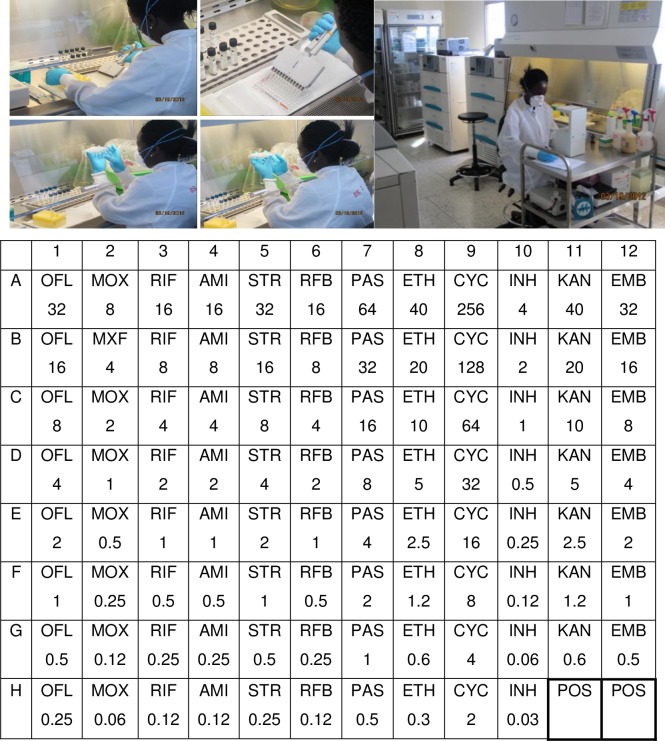
Shows personnel performing MYCOTB assay and the MYCOTB plate. The drugs showed in the table are; OFL, ofloxacin; MXF, moxifloxacin; RIF, rifampin; AMI, amikacin; STR, streptomycin; RFB, rifabutin; PAS, para-aminosalicylic acid; ETH, ethionamide; CYC, cycloserine; INH, isoniazid; KAN, kanamycin; EMB, ethambutol with their respective concetrations in *μ*g/mL and the positive control wells.

This was also done at the Mycobacteriology (BSL-3) laboratory of the Department of Medical Microbiology, Makerere University, and Kampala, Uganda. The MYCOTB plates were prepared, inoculated and reported as described elsewhere [6], and all performed according to the manufacturer’s instructions. Briefly, the inoculated MYCOTB plates were sealed and incubated at 37°C. The examination was done after 24 hours and 48 hours for contamination and thereafter 7, 10, 14 and 21 days. MICs were taken as the lowest concentration with no visible growth for a particular drug. The MYCOTB MIC breakpoints were; EMB 32 μg/ml, INH 4 μg/ml, RIF 4 μg/ml and STR 32μg/ml. As part of quality control, laboratory personnel were blinded to previous DST profiles and MYCOTB MIC testing and the MB7H10 reference comparator method testing were performed and interpreted by independent personnel. Fig 1. Shows the performance of MYCOTB plate DST and the MYCOTB plate with all possible drugs and the respective drug concentrations in μg/mL.

### Data analysis

For the current analysis, we considered LJ-DST or MB7H10 methods as a reference standard to MYCOTB assay, S1 File. Data were extracted and analysed using Stata version 13 (Stata Corp LP, 4905, Lakeway Drive College Station, TX, 77845, USA). For drugs with two concentrations, we considered data for the high concentration. For test agreement, we compared LJ DST versus MB7H10, LJ DST versus MYCOTB assay and MB7H10 versus MYCOTB assay. Sensitivity and specificity of MYCOTB plate and the respective 95% confidence intervals for each first line TB drug were calculated and compared using either reference standard.

### Ethics considerations

Ethical approvals for the main study were obtained from the School of Biomedical Sciences Research and ethics Committee (SBSREC), Makerere University and from the Uganda National Council of Science and Technology (UNCST). This was a laboratory based study with no access to patient information and therefore no informed consent was sought.

The individual in this manuscript has given written informed consent (as outlined in PLOS consent form) to publish these case details.

## Results

### Drug susceptibility testing patterns to first-line tuberculosis drugs

Of the 76 *M*. *tuberculosis isolates* tested, the number resistant were; 39 (51.3%) for streptomycin, 37 (48.7%) for isoniazid, 26 (34.2%) for rifampicin and 18 (23.7%) for ethambutol resistant. A total of 18 (23.7%) were resistant to both rifampicin and isoniazid (MDR-TB) on LJ-based DST.

**Fig 2:**
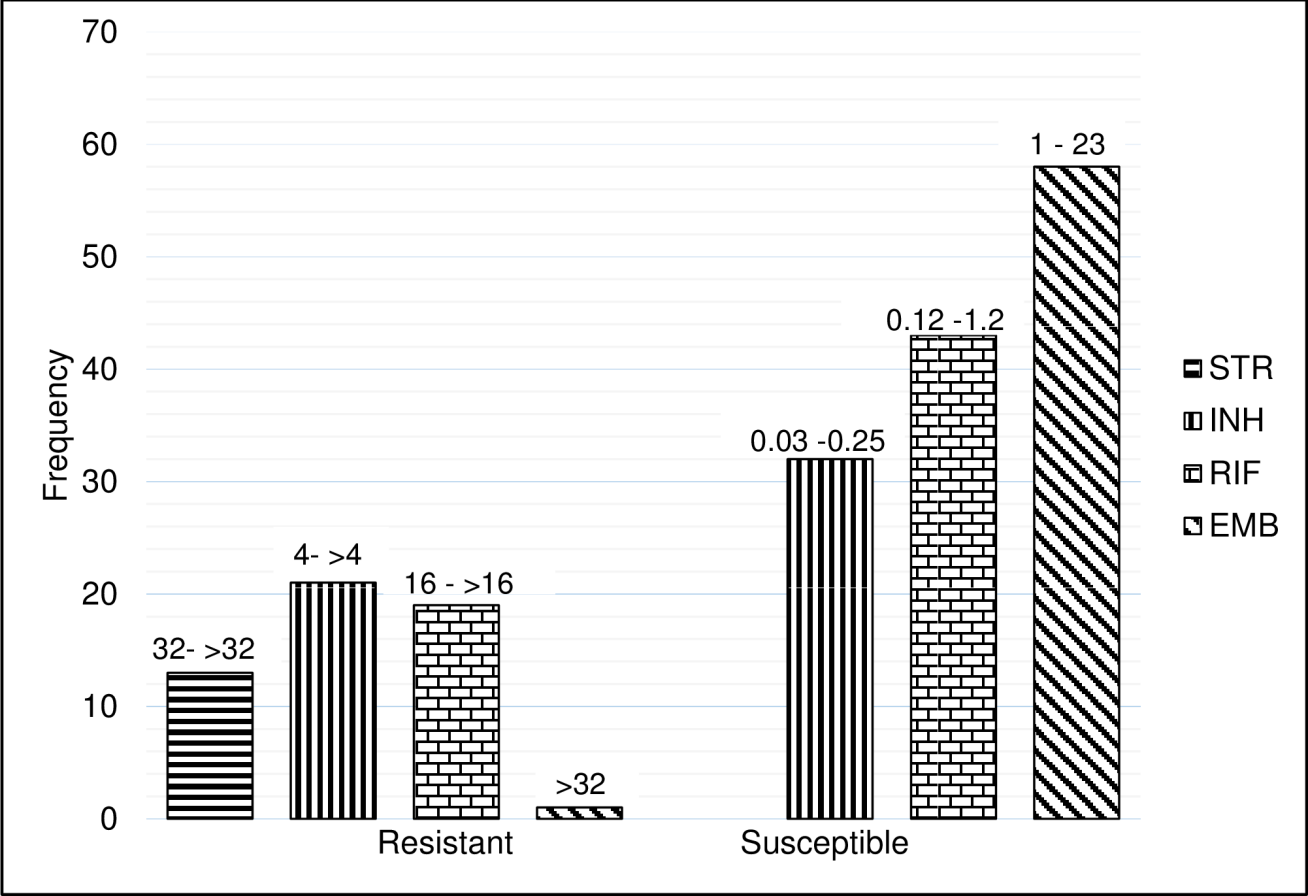
Shows *Mycobacterium tuberculosis* isolates resistant and susceptible by all methods with their corresponding MIC range per first line drug. STR = streptomycin, INH = isoniazid, RIF = rifampicin, EMB = ethambutol.

Isolates resistant by all methods were; streptomycin (n = 13), isoniazid (n = 21), rifampicin (n = 19) and ethambutol (n = 1) and susceptible by all methods to streptomycin (n = 0), isoniazid (n = 32), rifampicin (n = 43) and ethambutol (n = 58). Fig 2. illustrates the resistant and susceptible isolates by all methods (MB7H10, MYCOTB plate and LJ) with their MYCOTB MIC range per first-line drug.

### Agreement and comparative accuracy of MYCOTB plate with LJ and MB7H10 for first-line drug susceptibility testing

There was moderate agreement between LJ DST and MB7H10 for first line DST with highest agreement being for rifampicin (kappa 0.687) and lowest with ethambutol (kappa 0.082) which was also similar for MYCOTB plate compared with LJ DST, Table 1. The accuracy (sensitivity and specificity) of MYCOTB plate was lower when using LJ DST compared to MB7H10 as a reference method. Indeed, MYCOTB plate had good agreement with MB7H10 (kappa range 0.778–1.00) compared to LJ DST, Table 1.

**Table 1:** Agreement and comparative accuracy of MYCOTB plate with LJ and MB7H10 for first-line drug susceptibility testing.

Reference Method		MB7H10		MYCOTB plate		LJ/ MB7H10	LJ/ MYCOTB plate	MYCOTB plate	Reference method	MYCOTB plate		MYCOTB plate	MB7H10 /MYCOTB plate
LJ DST (μg/mL)	LJ DST	R	S	R	S	Kappa	Kappa	Sensitivity/ Specificity (95%CI)	MB7H10 (μg/mL)	R	S	Sensitivity/ Specificity (95%CI)	Kappa
STR (4.0)	R	13	26	14	25	0.275	0.300	87.5 (61.6–98.4)	STR (10)	15	0	93.8 (69.7–99.8)	0.959
	S	2	35	2	35			58.3 (44.8–70.9)		1	60	100	
INH (0.2)	R	15	12	22	15	0.498	0.496	75.9 (65.1–95.6)	INH (1.0)	25	7	96.2 (80.3–99.9)	0.778
	S	7	32	4	35			70.0 (55.3–82.1)		1	43	86.0 (73.2–94.1)	
RIF (40)	R	22	4	19	7	0.687	0.591	73.1 (52.2–88.4)	RIF (1.0)	26	3	100	0.915
	S	7	43	7	43			86.0(73.2–94.1)		0	47	94.0 (83.4–98.7)	
EMB (2.0)	R	1	17	1	17	0.082	0.082	100	EMB (10)	1	0	100	1.00
	S	0	58	0	58			77.3(66.2–86.2)		0	75	100	

R = resistant, S = Susceptible, CI = confidence interval, LJ = Lowenstein Jensen, MB = Middlebrook, MYCOTB = sensititre MYCOTB plate, STR = streptomycin, INH = isoniazid, RIF = rifampicin, EMB = ethambutol

### Discordance across the three methods for first-line *Mycobacterium tuberculosis* drug susceptibility testing

The discordance rates between the three DST methods were fewer with ethambutol (17) followed by rifampicin (18), isoniazid (24) and streptomycin (28). The corresponding MYCOTB plate MICs indicates a varied discordance pattern for the three methods. The MYCOTB plate agreed more with MB7H10 than with LJ-DST method, Table 2.

**Table 2:** Method discordances and the MYCOTB plate MICs.

Discordance	Frequency	MYCOTB plate MIC
**Rifampicin (40mg/ml)**		
LJr, MB7H10r, MYCOTBs	3	1–2
LJs, MB7H10r, MYCOTBs	4	0.12–1
LJr, MB7H10s, MYCOTBs	4	0.12–1β
LJs, MB7H10r, MYCOTBr	7	4 - >16*
LJr, MB7H10s, MYCOTBr	0	
LJs, MB7H10s, MYCOTBr	0	
**Isoniazid (1.0mg/ml)**		
LJr, MB7H10r, MYCOTBs	3	2
LJs, MB7H10r, MYCOTBs	3	2
LJr, MB7H10s, MYCOTBs	11	0.03–1ƚ
LJs, MB7H10r, MYCOTBr	7	>4
LJr, MB7H10s, MYCOTBr	0	
LJs, MB7H10s, MYCOTBr	0	
**Streptomycin**		
LJr, MB7H10r, MYCOTBs	0	
LJs, MB7H10r, MYCOTBs	0	
LJr, MB7H10s, MYCOTBs	25	0.25–16
LJs, MB7H10r, MYCOTBr	2	32, > 32
LJr, MB7H10s, MYCOTBr	1	>32
LJs, MB7H10s, MYCOTBr	0	
**Ethambutol**		
LJr, MB7H10r, MYCOTBs	0	
LJs, MB7H10r, MYCOTBs	0	
LJr, MB7H10s, MYCOTBs	17	0.5–16#
LJs, MB7H10r, MYCOTBr	0	
LJr, MB7H10s, MYCOTBr	0	
LJs, MB7H10s, MYCOTBr	0	

r = resistant, s = Susceptible, LJ = Lowenstein Jensen, MB = Middlebrook, MYCOTB = sensititre MYCOTB plate, MIC = minimum inhibitory concentration βMIC 1 (n = 1), 0.12 (n = 3)

*MIC 4 (n = 1), 16 (n = 1) and >16 (n = 5)

ƚMIC 4 (n = 1), 2 (n = 1) below 1 (n = 10), #MIC 16 (n = 1), 8 (n = 6) >8 (n = 10)

## Discussion

In this study we show that, using LJ-based DST as a reference standard, there is lower agreement with MB7H10 and MYCOTB plate for first line DST. We further show lower sensitivity and specificity of MYCOTB plate for first line DST when using LJ DST as a reference comparator yet the same was acceptably high when MB7H10 was used as a reference comparator. Our findings have both diagnostic and clinical management implications. Performing DST remains not only highly demanding in terms of infrastructure but also technically demanding. The low agreement between LJ DST and MB7H10, the two well-known and widely used reference methods presents a challenge in patient management as well as a challenge to diagnostic evaluation studies as to which of the two methods should be used as the gold standard. The high agreement between MB7H10 and MYCOTB plate indicates a performance gap in the commonly used LJ DST as the gold standard in many low-income countries (LIMC).

The higher sensitivity and specificity as well as agreement for the first line drugs on MYCOTB plate were documented in earlier studies which used MB7H10 as a reference standard [6,7]. An earlier study which compared LJ-DST and MYCOTB plate found high agreement [8] however did not compared with another solid DST method such as MB710-DST, presenting a unique component of our study.

Studies have documented a high prevalence of false resistance associated with streptomycin and ethambutol [4] attributing this to a small difference between the epidemiological cutoff (ECOF) and MIC [9,10,11,12,13] or borderline resistance. This is shown by our analysis of MICs among discordant results (Table 2) where the MICs for streptomycin and ethambutol widely vary and similar to previous study[4]. To date, reproducibility of results for streptomycin and ethambutol remains challenging justifying the need for a molecular-based diagnostic with well-correlated mutations which are known to have clinical implications. In settings where ethambutol still makes an important part of the continuation phase for the eight months regimen unlike in the six months regimen where rifampicin and isoniazid are used in the continuation phase, these findings of false ethambutol resistance have serious implications. For management of MDR-TB patients, this has implications since the number of first line drugs still susceptible to be carried forward will be reduced. The high agreement between MB7H10 and MYCOTB plate DST results and lower when compared with LJ-based DST points to a challenge between the gold standard phenotypic methods. The documented imperfect agreement between genotypic and phenotypic DST results also makes the choice of a tiebreaker limited and interpretation of the clinical implication of the reported discordance more challenging [14]. A recent study documented a reduction in unfavorable outcomes among rifampicin susceptible patients by treating, as isoniazid resistant, patients who had isoniazid susceptible results on solid media but with isoniazid-resistant results on liquid media [15].

Isoniazid can be resistant to either low or high drug concentration. This means that use of methods which offers limits of resistance such as MYCOTB plate are highly valuable in such situations as they enhance clinician’s judgment towards patient management. In agreement with earlier studies, we found high concordance between MB7H10, LJ and MYCOTB plate methods for rifampicin compared to isoniazid and streptomycin [6,7]. This indicates no major implications for settings with low MDR-TB rates as studies have attributed this discrepancy to disputed mutations in *rpoB* gene which confer low-level resistance to rifampicin. However, methods such as MYCOTB plate which offers MIC results could be necessary to generate such information so that the clinician can increase the dose or the treatment duration to eliminate such organisms. The fact that resistance due to such disputed mutations is less frequent, this poses less patient management implication in low MDR-TB settings.

The difference in agreements between MB7H10 and LJ-DST could be originating from the fact that MB7H10 has better *M*. *tuberculosis* yield compared to LJ [16]. This could mean that slow-growing resistant isolates may have been declared susceptible on LJ but resistant in MB7H10 and vice versa, a more yielding MB7H11 may lead to more discrepant results if compared with LJ-DST [16]. The MYCOTB plate uses broth-based media which is more yielding than solid media and this may also have led to more discordant results, when compared with LJ DST than with MB7H10, in case of slow growing strains. Guidelines on how to handle discordance between molecular and phenotypic DST results have been proposed [17], however, similar guidelines are needed in scenarios where phenotypic DSTs are discordant. Such scenarios may be possible when isolates tested using LJ-DST in the routine laboratory such as those from MDR-TB contact tracing studies are referred to the clinical research laboratory for further testing using MB7H10 based phenotypic DST method.

It is worth noting that the MYCOTB assay is a micro-titre plate based assay which demands more stringent protocols to prevent occupational exposure of laboratory personnel to MTB as well as to prevent contamination. This may be a limiting factor for MYCOTB roll-out in most of the resource limited settings where laboratory biosafety requirement are mostly inadequate and these challenges are similar to those in either LJ or MB7H10 DST methods. However in laboratories at intermediate or reference level with biosafety requirements, MYCOTB assay is feasible, easy to perform and interpret compared to LJ or MB7H10 DST methods.

Our findings may have been limited by the fact that LJ-DST were done at the NTRL and technical/ performance differences may have occurred, however, given the NTRL’s long-term experience in performing LJ-DST, we expect less technical errors in the LJ results to impact our conclusions. We were unable to have molecular results for the discordant assays yet MIC results from the MYCOTB assay for the discordant results provided more confidence for resistant or sensitive isolates to be true results. The sub-culturing of isolates for MB7H10 and MYCTB plate DST may have caused changes in the sample strain population from the original sample used for DST in presence of hetero-resistance [18]. Studies have indicated hero-resistance to affect DST results from the sub-cultured strains [19,20], however, this is expected to be minimal given the low prevalence of hetero-resistance. This is one of the few studies which have compared LJ and MB7H10 DST with MYCOTB plate and our findings are vital for future studies and recommendations.

## Conclusion

The performance of the two culture-based reference standards for phenotypic drug susceptibility testing varied much with wide range of MYCOTB plate MICs indicating both diagnostic and patient evaluation challenges. There was acceptable agreement and accuracy of MYCOTB plate for drug susceptibility testing when MB7H10 was used as reference stand than with LJ-DST. Results from MIC information makes the MYCOTB plate more suitable for guiding clinicians on the choice of the most appropriate TB treatment regimen as well as limits of detection for TB drug resistance. The results of isoniazid, streptomycin, and ethambutol should be considered for patient management with caution depending on the DST method and prevalence of resistance to such drugs.

## Supporting information

S1 File Supporting information file. Data set for LJ, MB7H10 and MYCOTB plate first-line drug susceptibility testing results (0 = susceptible and 1 = resistant).
(XLS)
